# Artificial Intelligence in Midwifery: A Scoping Review of Current Applications, Future Prospects, and Midwives’ Perspectives

**DOI:** 10.3390/healthcare13080942

**Published:** 2025-04-19

**Authors:** Paraskevi Giaxi, Victoria Vivilaki, Angeliki Sarella, Kleanthi Gourounti

**Affiliations:** Department of Midwifery, University of West Attica, Agioy Spyridonos 28, 12243 Egaleo, Greece; vvivilaki@uniwa.gr (V.V.); asera@uniwa.gr (A.S.); kgourounti@uniwa.gr (K.G.)

**Keywords:** artificial intelligence, maternal care, midwives, midwifery, prediction models, neonatal care, clinical decision making

## Abstract

**Background/Objectives**: Artificial intelligence (AI) is considered one of the core technological advancements of Industry 4.0, expected to transform various sectors, including healthcare. Midwifery can greatly benefit from AI; however, its current use, its future potential, and midwives’ attitudes remain underexplored. This study aimed to investigate the implementation of, prospects of, and attitudes of midwives toward AI. **Methods**: A scoping review was carried out, following the PRISMA guidelines. The search was conducted in Pubmed, Scopus, and Web of Science, from database inception to 2 February 2025. **Results**: Eight studies met the inclusion criteria. Although AI is not yet widely implemented in midwifery, it has notable potential. Several potential benefits were recorded, such as the enhancement of clinical education through personalized learning tools, such as AI-driven virtual patients and customized assessments, as well as a reduction in clinical errors via predictive models and real-time monitoring technologies. The adoption of AI is therefore expected to improve quality of care, particularly in perinatal and neonatal settings. However, it was found that the integration remains limited due to two key obstacles: ethical concerns (e.g., data privacy) and a notable level of anxiety or hesitation among midwives, associated with low levels of digital health literacy. **Conclusions**: It is important to form a relevant framework regarding the use of AI in midwifery, addressing ethical concerns and skepticism. Additionally, targeted educational interventions are needed to enhance midwives’ AI literacy and alleviate concerns. In general, it is essential to overcome these barriers to accelerate AI adoption in midwifery and unlock its full potential in perinatal care.

## 1. Introduction

Currently, humanity is on the edge of the Fourth Industrial Revolution, with artificial intelligence (AI) as its core advancement, expected to radically transform the way people interact, work, and, in general, live [1,2]. AI is the most recent technological development, distinct from the broader field of computer science and the evolution that has taken place in it from the emergence of computers to the present day. The key difference of artificial intelligence lies in its ability to critically evaluate data, learn from them, and make new decisions in an autonomous way based on its previous experience [2,3]. In addition, it has the ability to develop new thoughts, ideas, and solutions through data analysis, arriving at results different from those that would have been derived solely from the original set of information given to the system [4]. In this way, artificial intelligence seems capable of exceeding the limits of human ability to find solutions and solve problems, both in terms of speed and in terms of quality and efficiency [5].

As with any crisis, the COVID-19 pandemic prompted humans to seek new solutions in an adaptive manner in order to cope with the threatening situation they had to deal with. The development of AI was greatly accelerated during the pandemic period, due to the need to search for and identify the most effective drugs and vaccines more quickly [6]. Thus, there is an inextricable link between health sciences and the rapid advances in artificial intelligence in recent years. 

A reflection needs to be developed on whether health sciences can further capitalize on the progress of artificial intelligence. Indeed, it would be a real injustice if artificial intelligence, which evolved in response to the urgent need to meet health needs, were not to be further exploited in the healthcare sector. A key concern today is whether health systems can integrate and harness artificial intelligence into their normal operations and how they can make optimal use of it [7].

Midwifery is defined as the “profession of midwives” [8], a profession which, according to the WHO, is essential for maternal, newborn, and reproductive health [9]. The WHO supports that even though midwives and nurses constitute more than 50% of the healthcare workforce, in 55% of the WHO Member States there are less than 40 nursing and midwifery personnel per 100,000 population. The WHO supports that by 2030, the global shortage of midwives will worsen in developed countries [10]. Another related concern is the unequal distribution of technological advancements, such as AI, for midwives operating in different countries, which could increase inequality between health systems [11].

According to Eri et al. [12], there are different models in the way midwifery is delivered across the world. The first model is “Women-with-midwives: a model of interdependence”, developed in New Zealand and Scotland, which emphasizes reciprocity and the dynamic relationship between midwife and woman. The second one is the “model of exemplary midwifery practice” (USA), a model focusing on therapeutic outcomes, respectful and empowering care, and professional midwife identity. The third model is “carechildbearing women at high risk”, developed in Sweden, a model that prioritizes dignity, using a psychosocial approach. The fourth model is the “woman-centred childbirth model” (South Africa), a model with different phases from dependence to independence, promoting mutual participation and respect. The fifth model is the “primacy of the good midwife” (Iceland), emphasizing midwifery professionalism through caring, as well as through competence, wisdom, and personal development. The final model mentioned by Eri et al. [12] is “woman-centred childbirth care” (Sweden and Iceland), a model focusing on relationships, supportive birthing atmospheres, and the socio-cultural aspects of care.

Across the different models, midwives often face challenging working conditions (e.g., lack of proper training), highlighting the need to use new tools and methods to improve the professional practice in this discipline [9]. The use of AI in midwifery remains quite an important issue. According to O’Connor et al. [13], there is a big “distance” between the capabilities of AI in midwifery and its current impact. This technological advancement could reduce errors and save time, improving the quality of care, but, as they note, several problems, such as lack of training in the use of AI, could be responsible for the minimal positive effects of AI in the real world of midwifery practice.

To date, several organizations have focused on the potential impact of AI in healthcare. According to the OECD, AI can have a transformative impact in healthcare, since the related tools improve diagnostic precision, administrative tasks, and patient outcomes. The OECD supports that automatization will free up valuable time for health professionals to focus on important issues for patient care. However, the OECD mentions several ethical concerns, liability issues, data privacy issues, and the risk of algorithmic bias, which could have a negative impact on clinical decision making. In addition, the OECD mentions the lack of appropriate digital infrastructure and digital literacy as important problems regarding the adoption of AI in healthcare [14].

Regarding midwifery, relevant organizations have focused on the potential benefits and hazards of AI adoption. The Australian College of Midwives (ACM) has provided a written submission to the Select Committee on Adopting Artificial Intelligence, a relevant body established in March 2024 to help in the promotion and regulation of AI in the country. According to the ACM, there are basic priorities regarding legislation and midwife education regarding the use of AI. In addition, the concerns of the ACM are of utmost importance, since the ACM highlights the risks and harms arising from the adoption of AI. These concerns focus on duty overload for the staff at the stage of AI adoption, on lack of psychosocial interaction between midwives and mothers, and on the loss of human decision making [15]. Hence, apart from the obvious impact on efficiency, the ACM highlights several issues regarding the adoption of AI, which definitely have to be addressed.

Currently, it is important to carry out research regarding the adoption of AI in midwifery, in order to guide relevant policies. The WHO has highlighted this need, forming a strategy which consists of three specific pillars. The first is standards and governance, which require guidance and evidence-based research. The second is pooled investments. The third is sustainable implementation of AI in each country [16]. Taking the above as a starting point, carrying out a study regarding the current state of knowledge regarding AI adoption in midwifery is of utmost importance, in line with the first pillar mentioned by the WHO. This is extremely important in order to guide the necessary regulatory framework for AI adoption in midwifery. In that context, this study examines the issue of the utilization of artificial intelligence in the field of midwifery. In particular, this study examines the current use of artificial intelligence in midwifery and the perception of midwives toward its use. The final goal of this study is to come to conclusions regarding the future steps that have to be taken to improve the adoption of AI in midwifery.

## 2. Materials and Methods

### 2.1. Study Design

This study was a scoping review, aiming to investigate the general current status regarding AI in midwifery. The research followed a scoping review framework, which, as described by Arksey and O’Malley [17], involves five key stages: (1) identifying the research question, (2) identifying relevant studies, (3) study selection, (4) charting the data, and (5) collating, summarizing, and reporting the results. Following this structure, two specific research questions were formed: (a) What is the current state and future prospect of artificial intelligence adoption in midwifery? and (b) What are the perceptions of midwives regarding artificial intelligence integration into their profession?

### 2.2. Literature Search

A literature search was carried out in Pubmed, Scopus, and Web of Science, using the keywords (“artificial intelligence” OR AI) AND (midwifery OR midwives OR pregnancy OR maternal). The chronological criteria were from database inception to the 2nd of February 2025. Additionally, a snowball sampling approach was applied to identify relevant studies that may have been missed. This involved reviewing issues of related journals, examining reference lists of included studies, and searching other relevant publications. The selection process adhered to the PRISMA guidelines for systematic reviews [18]. The literature search process was carried out by the first and second authors.

### 2.3. Study Selection

For study selection, the inclusion criteria were as follows: (1) original research articles published in peer-reviewed journals, and (2) studies examining either the current state and/or future prospects of artificial intelligence applications in midwifery or the perceptions of midwives regarding the use of AI. The studies were excluded if (1) they were not published in English or (2) they were systematic reviews, scoping reviews, or meta-analyses. The identified abstracts were organized using Zotero version 6.0 for reference management software. The study selection process was carried out by the first and second authors, with disagreements resolved through the involvement of the third and fourth authors.

### 2.4. Data Extraction

Due to the different nature of the two research questions set in the study design process, different data were extracted for the first and for the second question. For the first question, the extracted data were the following: (1) authors and (2) current and future applications. These extracted data were decided due to the expected heterogeneity of those studies. For example, it was expected that some would be review studies and some research studies. Hence, a general and wide type of data extraction was considered essential (e.g., some studies would not have study participants). For the second question, the extracted data were the following: (1) authors, (2) country, (3) methodology, and (4) results. This type was chosen since it was expected that all these studies would be quantitative or qualitative. The first two authors carried out the data extraction process. Any potential disagreements were solved with the help of the third and fourth authors.

## 3. Results

### 3.1. Flow of Information

**Figure 1:**
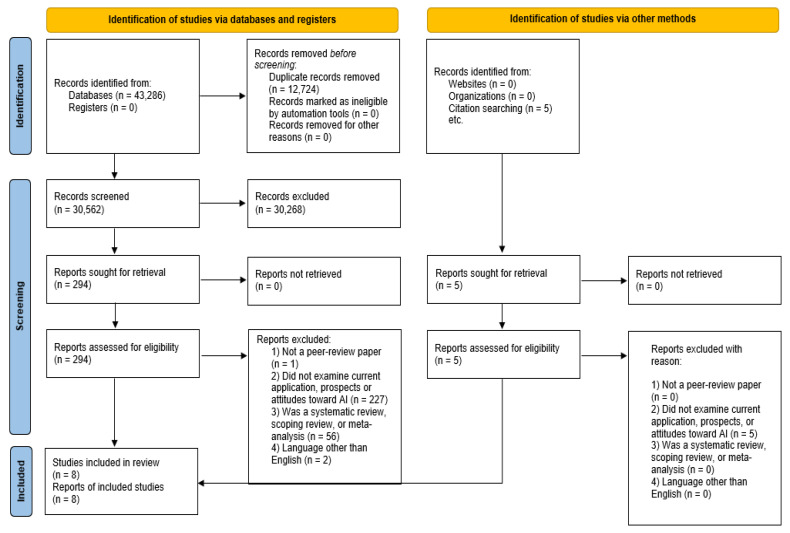
Selection process for the included studies.

The first step of the process was the identification of 43,286 records from the databases that were searched. Five additional records were identified from other sources, which were all ineligible since they did not examine current applications of, prospects of, or attitudes toward AI. For the records identified through databases, after removing the duplicates (n = 12,724), 30,562 records were screened. Next, 30,268 records were excluded, leading to 294 potentially relevant records. All 294 reports were successfully retrieved, and these studies were evaluated for their eligibility. At this step, 286 reports were excluded, since they did not meet the pre-set criteria. More specifically, they were excluded for not being peer-reviewed (1), not examining AI applications, prospects, or attitudes (227), being systematic/scoping reviews or meta-analyses (56), or being published in a language other than English (2). After excluding these records, eight studies met all eligibility criteria and were included in the narrative synthesis. The process from record identification to study inclusion is also presented in Figure 1.

### 3.2. Current State and Future Prospects

The question of the current state and future prospects of AI was the first question of this study. Based on the current knowledge, AI could have significant applications in midwifery. According to Irwin et al. [19], a potential contribution is the support of midwifery education. AI, including language models like ChatGPT (GPT-4, OpenAI), can lead to the development of personalized learning, through the generation of customized assessments, case studies, and interactive simulations. In this way, AI can make it possible for midwifery students to develop clinical skills in a controlled environment. As they noted in their paper, AI-driven virtual patients might enable midwifery students to practice decision making and patient care scenarios, avoiding the risks of real-case scenarios for human health. In addition, AI can support midwifery research, through real-time feedback, as well as through the development of automated assessments of writing tasks. The study by Irwin et al. [19] is particularly important due to the recognition of threats arising from that situation. More specifically, the authors raise concerns regarding academic integrity, artificial intelligence bias, and the ethical implications of artificial intelligence use in midwifery. In that context, their concluding remarks emphasize finding the way in which the use of artificial intelligence will be useful for midwives and patients.

In neonatal medicine, the potential role of artificial intelligence has been analyzed in a related paper by Kwok et al. [20]. In this paper, they highlighted some advancements in disease prediction, risk stratification, neurological diagnostic support, and image recognition technologies. For example, applications have been developed to improve the prediction of neonatal mortality and morbidity, particularly through machine learning models. These models are capable of detecting problems such as bronchopulmonary dysplasia and late-onset sepsis. In addition, artificial intelligence is helpful in pattern analysis, applied in large datasets, in order to support the understanding of nutrition’s impact on neonatal outcomes and to optimize vital sign monitoring. Real-time artificial intelligence applications in intensive care units can help continuously monitor patients, reduce false alarms, and allow earlier detection of critical conditions (e.g., respiratory failure) before they become evident on a clinical level. However, the aforementioned advancements remain at the research level and have not been widely adopted in neonatal care.

Another relevant study was published by Beam et al. [21]. As they stated, AI will transform the way neonatal intensive care units operate, leading to new ways to harness the vast amount of data generated by critically ill newborns. As data collection methods keep improving, AI is able to enhance and support clinical decision making, leading to better outcomes. In their paper, they identify four major areas of AI application: the interpretation of medical images, the ability of AI to predict health risks through the analysis of data stored in electronic health records, the integration of real-time monitoring data, and the streamlining of administrative tasks. More specifically, when analyzing images, AI can improve the accuracy of diagnosis, while predictive models can help the early detection of neonatal complications, in order to allow more timely interventions. Real-time monitoring saves time, since it leads to fewer unnecessary alarms, while also helping in the detection of changes in a neonate’s condition before it turns critical. In addition, automated processes in documentation, supported through AI, can lead to a significant reduction in administrative burdens, providing more time to focus on patients’ care.

The role of AI can also be regarded as important in health promotion. Georgakopoulou and Diamanti [22] explored its potential contribution in smoking cessation during pregnancy, focusing on its potential to overcome the limitations that traditional smoking cessation programs frequently face. The role of AI can be important, due to its ability to personalize interventions by predictive analytics in order to evaluate a wide range of individual risk factors, smoking behaviors, and support systems, thereby enhancing intervention efficacy. Related applications using AI could provide real-time support, such as, for example, personalized motivational messages. In addition, through AI, health professionals could have more accurate information about the current progress of women participating in such smoking cessation programs. However, Georgakopoulou and Diamanti [22] also acknowledge issues such as privacy concerns and the need to train health professionals in the use of AI. In general, this paper emphasizes the prospects of AI in such smoking cessation programs, although there is no direct reference on current uses of AI in smoking cessation programs during pregnancy.

The four studies mentioned above investigate both benefits and challenges associated with the possible adoption of AI in midwifery. Notably, in all these studies the authors rely on predictions or express thoughts, instead of relying on conclusions drawn from pilot programs or large-scale applications of AI in midwifery. Of note, Beam et al. [21] analyze similar prospects for the potential use of AI to those outlined by Kwok et al. [20], even though there is a time frame of two years between those two papers. Hence, AI in midwifery continues to be placed as a future prospect, instead of a well-established practice used in midwifery. The key findings of each study examined based on the first research question are presented in Table 1.

### 3.3. Midwives’ Perspectives

The second research question focused on midwives’ perspectives. To date, several studies have focused on the viewpoint of midwives toward the adoption of AI (Table 2). A relevant study with the use of questionnaires conducted in Turkey explored the association between AI readiness and related anxiety. The sample of the study consisted of midwifery (N = 240) and nursing students (N = 240), in order to examine how AI-related factors, such as knowledge, daily use, perceived occupational threat, and trust, are associated with AI readiness and anxiety. No significant differences were found between the two groups for AI readiness (*p* = 0.082) or anxiety (*p* = 0.486). The levels of readiness were significantly predicted by related knowledge and its use in daily life. As for AI anxiety, the use of AI in daily life, a high occupational threat perception, and low levels of trust were significantly associated with increased anxiety [23]. The study results regarding the second research question are presented in the following table.

A second relevant study was also carried out in Turkey, in order to examine the relationship between individual innovativeness levels and attitudes toward artificial intelligence among nursing and midwifery students. The sample of the study consisted of 500 midwifery and nursing students, who were administered various self-reported instruments (Personal Information Form, the Individual Innovation Scale, and the General Attitudes toward Artificial Intelligence Scale). The data of this study were collected in November and December 2023. The participants in the study had an inadequate level of individual innovativeness and generally positive perceptions concerning the use of AI. There was a positive association between individual innovativeness and attitudes toward artificial intelligence (*p* < 0.001), meaning that those with higher levels of innovativeness tended to have more favorable perceptions regarding the use of artificial intelligence. The attitudes toward artificial intelligence were significantly predicted by individual innovativeness (*p* < 0.001) [24].

Another relevant study was carried out in Turkey by Unlu Bidik [25], who examined the theoretical and clinical learning experiences and expectations of midwifery students using ChatGPT in their education. This qualitative study was based on the Heideggerian hermeneutic phenomenological approach in order to study the perspectives of 17 midwifery students. All participants were active users of ChatGPT. The data collection process was carried out through one-on-one, in-depth interviews using a structured three-part interview guide. The analysis of the data was carried out through content analysis, leading to three major themes, which focused on the role of ChatGPT in midwifery education, its impact on student development, and concerns regarding its use. More specifically, through the data analysis it was found that the study participants made use of ChatGPT mainly in order to enhance their theoretical knowledge, to facilitate clinical learning, and to receive support in academic tasks, such as assignments and research. The study participants had a positive attitude toward the time-saving and accessibility aspects of ChatGPT. However, they had serious concerns about its accuracy, the lack of proper referencing, and occasional inconsistencies in the responses that were provided. In addition, the participants of the present study had some concerns about data privacy and the potential ethical issues associated with the content created by ChatGPT. Of note, some of them supported that reliance on ChatGPT might lead to a significant reduction in interpersonal interactions with faculty and peers.

The final study included was the one by Çitil and Çitil Canbay [26], which was also carried out in Turkey. In this study, 18 midwives working in a public hospital were examined through semi-structured interviews as to their perceptions regarding AI. The interview consisted of nine open-ended questions designed to elicit their views on AI applications in midwifery care. The data of the studies were analyzed through content analysis. The data analysis led to three main themes, which were the following: (1) expectations, (2) prejudices, and (3) concerns. The study participants expected AI to reduce their workload, to improve time management, and to enhance the quality of midwives’ care. Nevertheless, the participants in the study were skeptical regarding its ability to replicate human competencies (e.g., personalized care and empathy). Many raised concerns about trust, ethical implications, and the potential dehumanization of childbirth. In conclusion, this study highlighted a general reluctance among midwives to adapt AI as a replacement for human care, raising several concerns.

In general, a common finding in the studies included in this part of the scoping review is the generally positive attitude that midwives have toward the use of artificial intelligence, particularly due to its potential to enhance theoretical knowledge, clinical learning, and workload efficiency [25,26,27]. However, a closer examination of the included studies leads to the conclusion that there are significant concerns. These concerns are associated with artificial intelligence’s ability to replicate human competencies, such as empathy and personalized care, as well as ethical issues, data privacy issues, and the potential dehumanization of care [25,26,27]. Another challenge is AI-related anxiety. More specifically, while readiness was linked to knowledge and daily use of AI, anxiety was associated with perceived occupational threats and low trust [23]. In addition, individual innovativeness had a positive impact on attitudes to the use of AI, with more innovative individuals having a higher level of acceptance [24]. In spite of the generally positive attitudes, there is some reluctance among midwives and students to fully embrace AI as a replacement for human care, emphasizing the need for a balanced approach [25,26,27].

## 4. Discussion

The present study aimed to explore the current state of AI integration into midwifery practice, highlight future prospects, and present the perspectives of midwives themselves. Based on the above analysis, several key findings can be drawn. First, there is a significant gap between the current degree of integration of AI into midwifery practice and the prospects for the future. Indeed, in the studies reviewed, the future is described with optimism, given the significant potential for AI to substantially improve midwifery, for example, through the ability to monitor the health status of pregnant women in real time [21]. However, a particularly interesting finding is that no specific examples of practical applications of AI in midwifery are mentioned, but instead, only the potential benefits of such a technology are highlighted, placing its use in an indefinite future. Furthermore, when critically examining the studies included in this review, there is no differentiation of the studies based on chronological criteria. One would expect that this trend would be more evident in early research in the field, while in later studies artificial intelligence would have been put into practice already. However, in all cases, a general expectation of the benefits of AI continues to be reported, with no specific incorporation of AI into clinical practice.

From the review research studies examined, the outlook for the future seems particularly optimistic, although there are some points of concern. These points are mentioned in two of the four articles included in the review, namely the studies by Irwin et al. [19] and Georgakopoulou and Diamanti [22]. In each case, these concerns relate mainly to the ethics underlying the use of AI and, to a lesser extent, whether or not its application will be beneficial. This is a general concern in healthcare during the mid-2020s, where the need to address the ethical concerns of AI during the stage of its adoption by healthcare organizations is imperative [27,28]. In addition, this finding is in line with the concerns expressed by prominent organizations, such as the OECD, the WHO, and the ACM [14,15,16,17].

Through the research included in this review, a fuller and more comprehensive understanding of how the issue is perceived by midwives emerges. AI emerges as a tool that can enhance theoretical knowledge and efficiency in midwifery practice, providing capabilities that were not previously available, leading to improvements in service delivery. This is confirmed by the views of the midwives themselves, as recorded in the qualitative research by Çitil and Çitil Canbay [26].

Particularly interesting is the finding from this review regarding the association between anxiety toward the use of AI and the attitudes of midwives. According to the literature, among health professionals involved in the care of neonates, such as nurses, for example, a negative correlation between anxiety toward AI and the intention to adopt it is recorded [29]. It seems, therefore, that something similar applies in the case of midwives, leading to the finding of a broader association between this anxiety and the practices of health professionals. This could be attributed to the excessive workload during the stage of AI adoption, a concern mentioned by the ACM [15].

Based on the findings of the included articles, this study could lead to a broader theoretical model for the integration of AI into midwifery practice. AI is seen as a tool that could significantly improve the quality of service delivery, both by enhancing midwifery education and by reducing errors in midwifery, confirming the viewpoint of the OECD on the improvement of healthcare provision due to AI [14]. However, there is still a significant gap between the current situation and the ideal application and use of AI in midwifery practice. Anxiety toward the use of AI, and the ethical concerns that arise, are the main obstacles that make it difficult to bridge this gap.

Based on these findings, several recommendations for midwifery practice can be made. Firstly, policymakers and scientific organizations active in this field should develop a fruitful reflection on the ethics that should govern the use of AI in midwifery practice. What is needed at this stage is the development of an ethical framework or “contract” to be adopted by midwives’ associations and organizations in different countries, thus facilitating the application of AI by addressing the related ethical concerns. This could be carried out through similar frameworks across countries, which according to the WHO is extremely important for AI regulation [16]. Moreover, overcoming these ethical challenges is also a broader necessity in the health sciences sector, which will enable AI to be adopted more rapidly and effectively in a variety of fields [30,31,32].

A second recommendation highlights the appropriate education and training of midwives. In the past, there was a similar concern about the adoption of modern technology in midwifery practice, as the adoption of computer technology was accompanied by high levels of anxiety [32]. This situation appears to be similar to the current situation, as the introduction of AI creates uncertainty and concern for health professionals. In the past, increasing midwives’ knowledge of new technologies was a necessary condition for reducing the anxiety they experienced [33,34]. Similarly, today it is considered necessary to develop education and training programs in AI, developed and delivered by the academic institutions themselves. Indeed, there is a significant difference between the informal use of tools such as ChatGPT by midwives to search for information and the systematic teaching of the operation and applications of AI as part of their curricula. Incorporating such technologies into education could help reduce anxiety and allow more effective adoption of AI in midwifery practice. This suggestion is in line with the needs highlighted by the ACM, which raise some important concerns about midwives’ AI-related literacy [15]. These programs have to be equally distributed to midwives in developed and developing countries, to prevent worsening inequality due to AI, which is already a significant point of concern [35].

A third recommendation is the training of AI models. As supported by Pavlik et al. [36], a major concern is the inadequate training of AI models. Any bias or errors during the model’s training leads to inaccuracies and mistakes while performing certain tasks. For this reason, collaboration needs to be developed between organizations regarding data sharing and algorithms’ training, leading to more accurate and effective models.

In general, this progress will help us move toward a new model of care in midwifery, the AI-powered model of care. There will therefore be a seventh model apart from those mentioned by Eri et al. [12], as a result of the technological progress of the Fourth Industrial Revolution. Addressing the ethical and practical challenges of AI applications is essential to increase the level of AI adoption in midwifery and to ensure that this model will improve the quality of care and the working conditions of midwives.

In any case, the present scoping review faces two important limitations, which should be noted. First, the number of articles that met the criteria for inclusion in the review was relatively limited, which affects the scope of the findings. Second, all of the research studies included in the review were conducted in one specific country, Turkey. This may be due to the particular research interest that has developed in this country regarding the adoption of artificial intelligence in midwifery practice. However, this geographical dimension is a factor that reduces the generalizability of the findings of this review to other contexts and countries. For these reasons, it becomes imperative to conduct further research that will examine the adoption of artificial intelligence in midwifery practice in different cultural and healthcare settings, thus allowing for a more comprehensive understanding of the issue.

In addition, some directions for future research could be provided by information that has not been found through the articles examined, although such information is essential for the improvement of AI adoption in midwifery. More specifically, research should investigate what safeguards are in place to prevent AI-based decision making from reinforcing existing disparities in maternal healthcare, searching for relevant regulatory frameworks. This systematic review focused on published papers, not on material from specific organizations, where such information could be available. Further, it is important to examine what feedback mechanisms have been developed so that midwives can contribute to the improvement of AI in midwifery, as well as what mechanisms have been developed for collaboration between different institutions. Moreover, it is necessary to further emphasize studying the ethical concerns of midwives, since these concerns could debar the further adoption of AI. Finally, it is important to further study the role of digital literacy regarding artificial intelligence, which could have a significant impact on midwives’ ability to use AI.

## 5. Conclusions

Through this scoping review, it was found that artificial intelligence could have significant positive effects on midwife practice, with the function of better preparation of midwives for their clinical work, as well as due to the possibility of its practical utilization in order to avoid errors and improve the quality of services provided. The main obstacles relate to the intense anxiety of midwives toward the use of artificial intelligence and the ethical concerns that arise. Consequently, action is needed to address these two barriers, which involves developing educational interventions to improve midwives’ AI literacy. Finally, future research is warranted to better understand and monitor the adoption of artificial intelligence in the field of midwifery.

## Figures and Tables

**Table 1:** The extracted data for the current state and future prospects of AI in midwifery.

Study	Positives/Benefits of AI	Negatives/Threats of AI
Irwin et al. (2023) [19]	Supports midwifery education through personalized learning, AI-driven virtual patients, real-time feedback, and automated assessment.	Raises concerns about academic integrity, AI bias, and ethical implications in midwifery education.
Kwok et al. (2022) [20]	Enhances disease prediction, risk stratification, and real-time monitoring in neonatal care, enabling early detection of conditions like bronchopulmonary dysplasia and late-onset sepsis.	At an early research stage; ethical concerns regarding data privacy and AI decision making.
Beam et al. (2024) [21]	Improves medical imaging interpretation, predicts health risks via electronic health records, enhances real-time monitoring, and streamlines administrative tasks in neonatal intensive care units.	Could reduce human judgment; ethical concerns regarding the reliability of AI decision making.
Georgakopoulou and Diamanti (2024) [22]	Personalizes smoking cessation interventions during pregnancy through predictive analytics and real-time support, providing better tracking of patient progress.	Privacy concerns and the lack of training for professionals in AI usage.

**Table 2:** The extracted data of studies on midwives’ attitudes toward AI.

Authors	Country	Methodology	Results
Demir-Kaymak et al. (2024) [23]	Turkey	Quantitative study with questionnaires for midwifery (N = 240) and nursing students (N = 240).	No significant differences in AI readiness (*p* = 0.082) and AI anxiety (*p* = 0.486) between groups. AI readiness predicted by AI knowledge and daily use. AI anxiety linked to daily use, high occupational threat perception, and low trust.
Erciyas et al. (2024) [24]	Turkey	Quantitative study with questionnaires for 500 midwifery and nursing students using surveys.	Participants had inadequate individual innovativeness but positive attitudes toward AI. Positive association between innovativeness and AI attitudes (*p* < 0.001). Attitudes predicted by individual innovativeness (*p* < 0.001).
Unlu Bidik (2025) [25]	Turkey	Qualitative study with interviews using Heideggerian hermeneutic phenomenology (N = 17 students).	ChatGPT used for theoretical knowledge, clinical learning, and academic tasks. Positive perceptions of time saving and accessibility, but concerns about accuracy, referencing, data privacy, and reduced interpersonal interactions.
Çitil and Çitil Canbay (2022) [26]	Turkey	Qualitative study with semi-structured interviews (N = 18 midwives).	Themes: expectations (workload reduction, improved care), prejudices (skepticism about AI replicating human skills), and concerns (trust, ethics, dehumanization). Reluctance to replace human care with AI.

## Data Availability

Not applicable.

## Abbreviations

The following abbreviation is used in this manuscript:

AI	Artificial intelligence
